# Harnessing X‐Ray Energy‐Dependent Attenuation of Bismuth‐Based Nanoprobes for Accurate Diagnosis of Liver Fibrosis

**DOI:** 10.1002/advs.202002548

**Published:** 2021-03-16

**Authors:** Shiman Wu, Xianfu Meng, Xingwu Jiang, Yelin Wu, Shaojie Zhai, Xiaoshuang Wang, Yanyan Liu, Jiawen Zhang, Xinxin Zhao, Yan Zhou, Wenbo Bu, Zhenwei Yao

**Affiliations:** ^1^ Department of Radiology Huashan Hospital Fudan University Shanghai 200040 P. R. China; ^2^ Department of Materials Science Fudan University Shanghai 200433 P. R. China; ^3^ Tongji University Cancer Center Shanghai Tenth People's Hospital Tongji University School of Medicine Shanghai 200072 P. R. China; ^4^ State Key Laboratory of High Performance Ceramics and Superfine Microstructure Shanghai Institute of Ceramics Chinese Academy of Sciences Shanghai 200050 P. R. China; ^5^ Department of Radiology Renji Hospital School of Medicine Shanghai Jiao Tong University Shanghai 200127 P. R. China

**Keywords:** BiF_3_@PDA@HA nanoprobes, hyaluronic acid, liver fibrosis, spectral computed tomography

## Abstract

Timely detection of liver fibrosis by X‐ray computed tomography (CT) can prevent its progression to fatal liver diseases. However, it remains quite challenging because conventional CT can only identify the difference in density instead of X‐ray attenuation characteristics. Spectral CT can generate monochromatic imaging to specify X‐ray attenuation characteristics of the scanned matter. Herein, an X‐ray energy‐dependent attenuation strategy originated from bismuth (Bi)‐based nanoprobes (BiF_3_@PDA@HA) is proposed for the accurate diagnosis of liver fibrosis. Bi element in BiF_3_@PDA@HA can exhibit characteristic attenuation depending on different levels of X‐ray energy via spectral CT, and that is challenging for conventional CT. In this study, selectively accumulating BiF_3_@PDA@HA nanoprobes in the hepatic fibrosis areas can significantly elevate CT value for 40 Hounsfield units on 70 keV monochromatic images, successfully differentiating from healthy livers and achieving the diagnosis of liver fibrosis. Furthermore, the enhancement produced by the BiF_3_@PDA@HA nanoprobes in vivo increases as the monochromatic energy decreases from 70 to 40 keV, optimizing the conspicuity of the diseased areas. As a proof of concept, the strategically designed nanoprobes with energy‐dependent attenuation characteristics not only expand the scope of CT application, but also hold excellent potential for precise imaging‐based disease diagnosis.

## Graphical abstract

A strategy of energy‐dependent attenuation based on bismuth (Bi) nanomaterials is proposed. BiF_3_@PDA@HA nanoprobes accumulated in fibrosis‐afflicted areas can attenuate characteristically depending on monoenergetic X‐ray, further leading to fibrotic livers conspicuous via spectral computed tomography (CT). Yet conventional CT with polyenergetic X‐ray can't distinguish liver fibrosis from healthy liver.

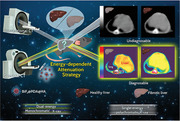

## 1 Introduction

Liver cirrhosis and hepatocellular carcinoma (HCC) are responsible for approximately two million deaths annually worldwide due to the lack of effective treatment options.^[^ ^1^ ^]^ Cirrhosis and HCC commonly result from chronic liver fibrosis.^[^ ^2^ ^]^ Fortunately, fibrotic progression in liver can be reversed if it is diagnosed in a timely manner,^[^ ^3^ ^]^ further improving the patients’ quality of life and survival rate.^[^ ^4^ ^]^ However, the routine diagnosis of liver fibrosis depends on highly invasive biopsies that lead to pain and other complications.^[^ ^5^ ^]^ Therefore, there is an urgent need to exploit novel imaging techniques for accurately diagnosing liver fibrosis in a non‐invasive manner.^[^ ^6^ ^]^

X‐ray computed tomography (CT) is a common diagnostic tool for tumors,^[^ ^7^ ^]^ internal injuries,^[^ ^8^ ^]^ fractures,^[^ ^9^ ^]^ and so on. However, since conventional CT uses a polychromatic (polyenergetic)^[^ ^10^ ^]^ X‐ray spectrum with multienergy photons,^[^ ^11^ ^]^ it can only distinguish materials based on their difference in density rather than attenuation characteristics.^[^ ^12^ ^]^ Hence, conventional CT cannot differentiate between the fibrotic and healthy liver tissues due to their similarity in density. Acquired with high‐energy and low‐energy X‐ray settings, spectral CT is capable of distinguishing different kinds of tissue in vivo based on their attenuation characteristics^[^ ^13^ ^]^ and generates monochromatic (monoenergetic) X‐ray images that represent single X‐ray photon energy level ranging from 40–140 keV.^[^ ^14^ ^]^ Yet, when exploiting spectral CT to detect liver fibrosis, a challenge remains: fibrotic and healthy livers share similar X‐ray attenuation coefficients. To better distinguish abnormality in soft tissue, clinical CT scans commonly require the administration of iodine‐based contrast agents.^[^ ^15^ ^]^ Nevertheless, the clinical X‐ray spectra contain few photons around the K‐edge of iodine (33 keV),^[^ ^16^ ^]^ leading to an exceedingly weak characteristic attenuation of iodine to be identified by spectral CT. Furthermore, the disadvantages of clinical iodinated small‐molecule agents, including short metabolic time^[^ ^17^ ^]^ and incompetence of disease targeting,^[^ ^18^ ^]^ make iodine‐enhanced spectral CT still inadequate to distinguish between the fibrotic and healthy livers. Bismuth (Bi) has a K‐edge value of 90.5 keV,^[^ ^19^ ^]^ and large X‐ray attenuation coefficient (5.74 cm^2^ g^−1^ at 100 keV).^[^ ^20^ ^]^ Therefore, Bi‐based nanoparticles can overcome the shortcomings of iodinated small molecules, offering higher‐performing contrast enhancement on CT imaging.^[^ ^21^ ^]^ More importantly, Bi with high atomic number (Z), can show characteristic attenuation at different energies of the X‐ray beam, known as “energy‐dependent attenuation,”^[^ ^22^ ^]^ a property that is suitable for spectral CT. Thus, it is of considerable significance to reasonably design Bi‐based nanoprobes for achieving accurate diagnosis of liver fibrosis.

**Scheme 1:**
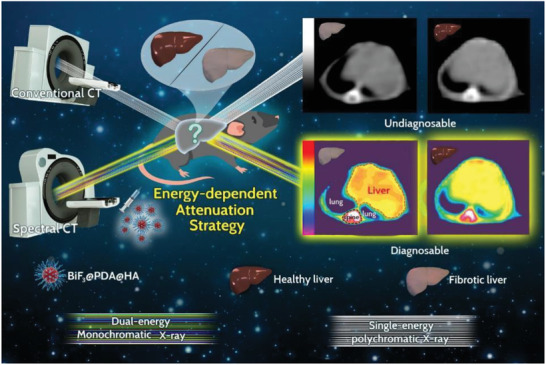
Schematic diagram of the energy‐dependent attenuation strategy for diagnosing liver fibrosis. After injecting BiF_3_@PDA@HA nanoprobes, conventional CT and spectral CT were performed in liver fibrosis model mice and healthy control mice. Bismuth (Bi) has characteristic X‐ray attenuation depending on different X‐ray photon energy, also known as energy‐dependent attenuation. As spectral CT can generate monoenergetic images representing single energy level of X‐ray, the strategically designed BiF_3_@PDA@HA nanoprobes can show unique enhancement in fibrotic liver corresponding with certain energy level of X‐ray via spectral CT, achieving the diagnosis of liver fibrosis. But the conventional CT with polychromatic X‐ray cannot reveal the characteristic energy‐dependent attenuation of Bi, thus incapable of detecting liver fibrosis.

Herein, an innovative X‐ray energy‐dependent attenuation strategy was proposed for the precise diagnosis of liver fibrosis. Liver fibrosis is characterized by the excessive proliferation and abnormal deposition of the extracellular matrix proteins, such as collagen and hyaluronic acids (HA).^[^ ^23^ ^]^ Thus, HA is considered as a vital biomarker of liver fibrosis, and significantly correlated with the severity of liver fibrosis.^[^ ^24^ ^]^ By conjugating HA to Bi‐based nanoparticles, novel liver‐fibrosis‐targeting contrast nanoagents were prepared for diagnosing liver fibrosis via spectral CT. The targeting performance of HA toward liver fibrosis has been confirmed by multiple previous studies.^[^ ^25^ ^]^ Thus, HA modification can enable BiF_3_@PDA@HA nanoprobes to selectively accumulate in the fibrotic tissues as opposed to healthy liver tissues, allowing differential imaging between the healthy and fibrotic livers by spectral CT. In contrast, conventional CT was incompetent (**Scheme** **1**). Specifically, on the 70 keV monoenergetic images of spectral CT, liver CT Hounsfield unit (HU) value manifested approximately onefold elevation (from 38.50 ± 3.63 to 79.67 ± 11.57 HU) in mice model of liver fibrosis at 30 min after injection of BiF_3_@PDA@HA nanoprobes. Furthermore, the most obvious distinction between the healthy and fibrotic livers was achieved at the X‐ray energy of 40 keV. Taken together, the X‐ray energy‐dependent attenuation characteristic of BiF_3_@PDA@HA was quite qualified for the non‐invasively precise diagnosis of liver fibrosis via spectral CT, confirming the feasibility of energy‐dependent attenuation strategy.

## 2 Results and Discussion

### 2.1 Synthesis and Characterization

**Figure 1:**
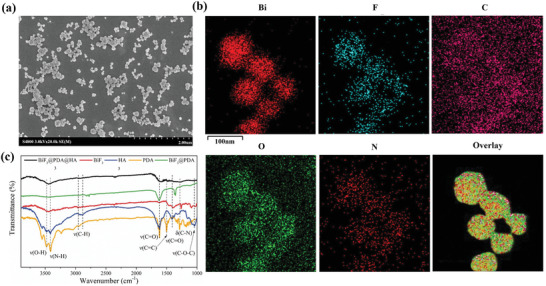
Characterization of BiF_3_@PDA@HA nanoprobes. a) Scanning electron microscopy (SEM) images of BiF_3_@PDA@HA. b) Element mappings (Bi, F, C, O, and N) of BiF_3_@PDA@HA. c) Fourier transformation infrared spectra (FT‐IR) of BiF_3_@PDA@HA, BiF_3_@PDA, BiF_3_, HA, and PDA.

BiF_3_ nanoparticles were synthesized as previously described,^[^ ^26^ ^]^ and sequentially modified with dopamine (DA) and HA for hydrophilicity and liver‐fibrosis‐targeting, respectively. Transmission electron microscopy (TEM) images (Figure S1, Supporting Information) confirmed the successful preparation of BiF_3_ nanoparticles and BiF_3_@PDA@HA nanoprobes. Scanning electron microscopy (SEM) demonstrated the uniform spherical morphology of both BiF_3_ nanoparticles (Figure S2, Supporting Information) and BiF_3_@PDA@HA nanoprobes (**Figure** **1**a). Also, BiF_3_@PDA@HA nanoprobes showed excellent hydrodynamic size distribution centered at 170.7 nm (Figure S3, Supporting Information), explaining their high dispersity without obvious aggregation. Elemental mapping (Figure 1b) further revealed the presence of Bi, F, C, O, and N element in the BiF_3_@PDA@HA nanoprobes. Furthermore, Fourier transform infrared (FT‐IR) spectra verified the successful surface modification with polydopamine (PDA) and HA (Figure 1c). Finally, X‐ray diffraction (XRD) showed that BiF_3_ nanoparticles, BiF_3_@PDA nanoparticles, and BiF_3_@PDA@HA nanoprobes were in cubic phase (Figure S4, Supporting Information).

### 2.2 Computed Tomography Imaging of Bismuth‐Based Nanoprobes In Vitro

**Figure 2:**
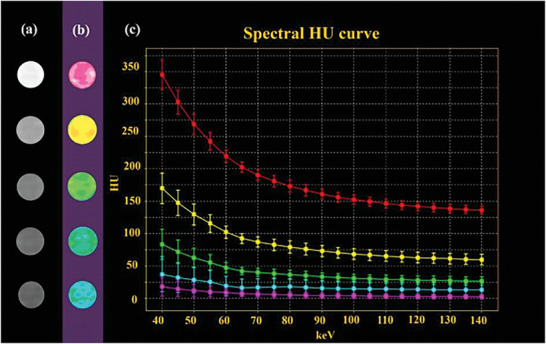
CT performance of BiF_3_@PDA@HA nanoprobes in vitro. a) Conventional CT images, b) spectral CT monochromatic images, and c) spectral HU curve of BiF_3_@PDA@HA nanoprobes with gradiently descent Bi concentrations (10 000, 5000, 2500, 1250, and 625 µg mL^−1^, from top to bottom) with normalization (CT water = 0 HU)

The imaging ability of BiF_3_@PDA@HA nanoprobes with gradient Bi concentration was determined by spectral CT and conventional CT. As shown in **Figure** **2**, CT phantom images for BiF_3_@PDA@HA nanoprobes of the higher Bi concentrations were brighter in both forms of CT. But the nanoprobes with the lower amount of Bi became more indistinguishable in conventional CT images (Figure 2a). However, the disparity among different concentrations of Bi was more apparent in spectral CT, even in the range of low Bi concentration (Figure 2b). Furthermore, as the spectral HU curves shown, the CT value of BiF_3_@PDA@HA nanoprobes increased sharply as the monochromatic energy of spectral CT decreased (Figure 2c). But the spectral HU curves dropped down as concentration of Bi decreased. Spectral HU curve trend of each sample represents their individual characteristic,^[^ ^27^ ^]^ hence the energy‐dependent regularity shown in the spectral HU curve of Bi‐based nanoprobes could be used for identifying the existence of selectively accumulated Bi in vivo. Taken together, BiF_3_@PDA@HA nanoprobes could be used as spectral CT contrast agents by showing characteristic energy‐dependent attenuation.

### 2.3 Cellular Uptake of Bismuth‐Based Nanoprobes In Vitro

The biocompatibility of the nanoparticles was evaluated on the murine hepatocyte AML12 line. The targeted BiF_3_@PDA@HA nanoprobes and the untargeted BiF_3_@PDA@PEG nanoparticles were non‐toxic in terms of the cellular viability (Figure S5, Supporting Information).

**Figure 3:**
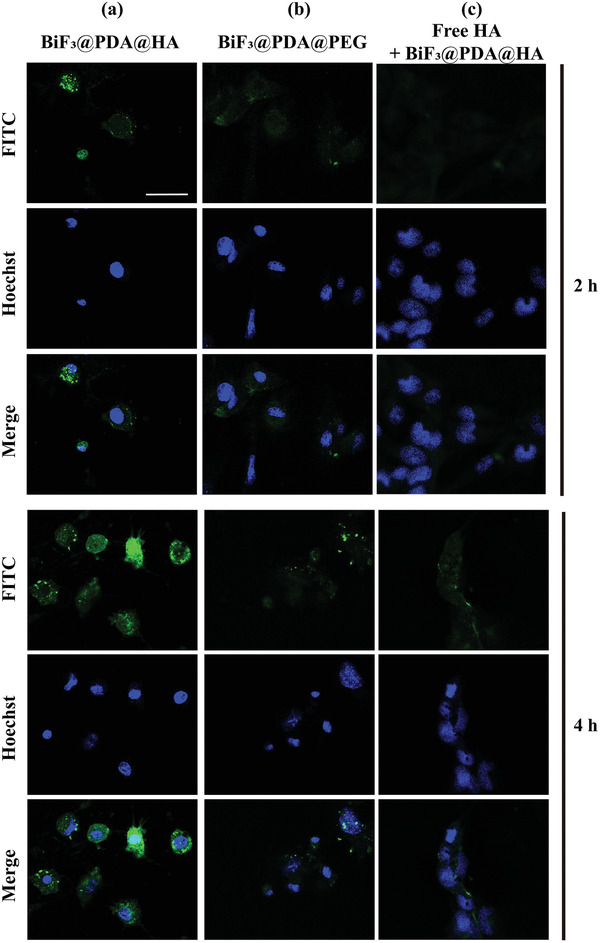
Representative CLSM images of cellular uptake of Bi‐based nanoprobes in vitro. a,b) HSCs incubated with BiF_3_@PDA@HA (a) and BiF_3_@PDA@PEG (b) for 2 and 4 h. c) After blocking the CD44 receptor with free HA (5 mg mL^−1^) for 1 h, the HSCs were incubated with BiF_3_@PDA@HA nanoprobes for 2 and 4 h. CLSM: confocal laser scanning microscopy; HSCs: hepatic stellate cells; HA: hyaluronic acids.

Furthermore, hepatic stellate cells (HSCs) as liver fibrosis hallmark cells,^[^ ^28^ ^]^ were used to confirm the successful target‐specific effect of BiF_3_@PDA@HA. HSCs activation is a key process in the liver fibrogenesis, and the CD44 receptor is highly expressed in HSCs, which is a major receptor of HA.^[^ ^29^ ^]^ From the observation through confocal laser scanning microscopy (CLSM), a higher degree of BiF_3_@PDA@HA nanoprobes was selectively internalized by HSCs than BiF_3_@PDA@PEG nanoparticles. As shown in **Figure** **3**, the BiF_3_@PDA@HA showed the higher green‐fluorescence intensity which corroborated with faster cellular uptake compared to non‐targeted nanoparticles, BiF_3_@PDA@PEG BiF_3_@PDA@HA nanoprobes were distributed across the cell surface and cytoplasm. In contrast, the untargeted BiF_3_@PDA@PEG and nanoparticles were rarely taken up by HSCs. Besides, the fluorescence intensity significantly decreased when excessive HA was added one hour before BiF_3_@PDA@HA, demonstrating similar fluorescence intensity with non‐targeted nanoparticles in a relatively low range. Taken together, selective accumulation of targeted BiF_3_@PDA@HA nanoparticles is probably due to outstanding affinity of HA‐ligand to CD44 receptor‐mediated endocytosis in HSCs, indicating that BiF_3_@PDA@HA nanoprobes could target HSCs effectively in vitro.

### 2.4 Computed Tomography Imaging of Bismuth‐Based Nanoprobes In Vivo

#### 2.4.1 Parallel Comparison between Gemstone Spectral Computed Tomography and Conventional Computed Tomography

**Figure 4:**
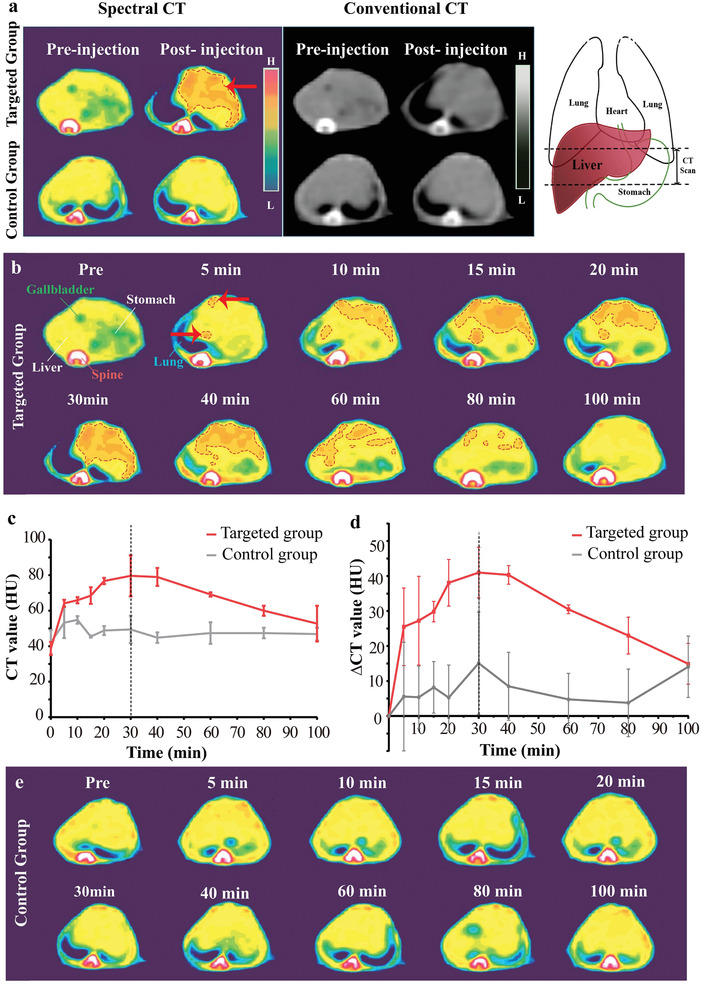
Computed tomography (CT) imaging of BiF_3_@PDA@HA nanoprobes in vivo. a) Representative CT images acquired at two time points (preinjection and 30 min after the injection of BiF_3_@PDA@HA) for two groups via spectral CT (70 keV) and conventional CT (140 kVp). Red arrow and red dashed circle represent the enhanced area. The schematic drawings illustrate the taken CT sections. b) Representative spectral CT images of the targeted group before and after the injection of BiF_3_@PDA@HA. c,d) Dynamic time courses of CT value and CT value change (ΔCT value) of hepatic parenchyma of targeted group (red line, *n* = 3) and control group (grey line, *n* = 3). Data was presented as mean ± standard deviation. e) Representative spectral CT images of control group before and after the injection of BiF_3_@PDA@HA.

The CT imaging ability was investigated by injecting BiF_3_@PDA@HA nanoprobes into mice with liver fibrosis (targeted group) and healthy mice (control group). As a monochromatic energy level of 70 keV in spectral CT is similar to the average photon energy of a polychromatic X‐ray beam produced by conventional CT,^[^ ^30^ ^]^ the images obtained from each form of CT were compared. On 70 keV monoenergetic images of spectral CT, the fibrotic liver in the targeted group manifested enhancement after the injection of BiF_3_@PDA@HA (**Figure** **4**a, red arrow, and red dashed area), while no apparent enhancement was shown in the control group. Besides, on conventional CT images, both groups exhibited similar performance in hepatic parenchyma post‐injection, with negligible differences from the images of preinjection.

Therefore, the dynamic change of the two groups on 70 keV monoenergetic images via spectral CT was investigated in detail. CT value of liver gradually increased and then decreased in the targeted group (Figure 4b, red dashed areas). Besides, the CT value was quantified for diagnosing the fibrosis sensitively. As shown in Figure 4c,d (red lines), liver CT value and ΔCT value (ΔCT value = CT_post_ − CT_pre_) of the targeted group increased gradually and reached to a peak at 30 min post‐injection (CT value: 79.67 ± 11.57 HU, ΔCT value: 41.00 ± 15.10 HU). The enhancement then began to weaken but sustained visibly until 80 min post‐injection. Predominant uptake of BiF_3_@PDA@HA nanoprobes were observed in liver from coronal projection (Figure S6, Supporting Information). In contrast, in the control group, no contrast‐enhanced region inside healthy liver tissue was observed (Figure 4e), or in other tissue following BiF_3_@PDA@HA injection (Figure S7, Supporting Information). Besides, CT value of the targeted group at each time point was statistically significantly higher than that of the control group from 5 to 80 min post‐injection (**Table** **1**). Taken together, the unique attenuation of Bi on 70 keV enabled spectral CT to identify the dynamic changes of Bi concentration in vivo and generated corresponding enhancement in targeted areas on monoenergetic images, further clearly distinguishing liver fibrosis from the healthy liver tissue. Utilizing spectral CT, the fibrosis‐afflicted areas could be successfully delineated, leading to the diagnosis of liver fibrosis.

**Table 1:** Mean CT value of three groups and multicomparison results

CT value [HU]	Targeted group	Control group	Non‐targeted group
0 min	38.75 ± 3.63	42.17 ± 6.88	37.92 ± 6.02
5 min	64.17 ± 1.94	53.25 ± 8.65^*^	41.08 ± 6.91^**^
10 min	65.92 ± 1.756	54.83 ± 2.08^*^	43.92 ± 1.23^**^
15 min	68.50 ± 4.68	45.42 ± 0.58^***^	38.83 ± 1.94^***^
20 min	76.75 ± 1.75	48.83 ± 2.504^***^	33.92 ± 7.715^***^
30 min	79.67 ± 11.57	49.42 ± 7.75^***^	40.42 ± 3.75^***^
40 min	79.00 ± 5.06	44.83 ± 2.93^***^	36.25 ± 3.31^***^
60 min	69.17 ± 1.18	47.42 ± 6.08^***^	38.58 ± 4.73^***^
80 min	60.00 ± 2.82	47.42 ± 2.96^*^	35.08 ± 2.27^***^
100 min	52.75 ± 10.04	46.83 ± 3.626	36.33 ± 1.44^**^

Note: “min” means minutes. Data were mean ± standard deviation (*n* = 3 for each group);

Determined by two‐way ANOVA, the liver CT value of all groups was significant for each factor: group (*p* < 0.0001), time (*p* < 0.0001), and group × time (*p* < 0.0001). Then the differences at each time point among groups were compared by Tukey's multiple comparison test;

*/**/*** indicated statistically significant difference from the targeted group (*p* < 0.05/0.001/0.0001).

Moreover, histopathological examination confirmed liver fibrosis in the targeted group. As shown in Figure S8, Supporting Information, the affected liver tissue had steatotic degeneration of hepatocytes, massive infiltration of inflammatory cells, and extensive fibrous deposition. In contrast, no pathological change was observed in healthy livers of the control group. In addition, abundant Sirius red‐stained collagen fibers were observed in the fibrotic livers of the targeted group and absent in healthy livers of the control group.

**Figure 5:**
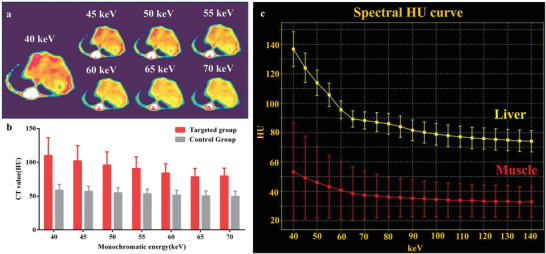
Energy‐dependent attenuation characteristics of BiF_3_@PDA@HA nanoprobes in vivo at 30 min post‐injection. a) 40–70 keV monochromatic images of the targeted group. b) Comparison between the targeted group (*n* = 3) and the control group (*n* = 3) in CT value of monochromatic images of 40–70 keV. Data was presented as mean ± standard deviation. c) Spectral HU curves of liver (yellow line) and muscle (red line) of the targeted group. CT: computed tomography.

Furthermore, changing the monochromatic energy of spectral CT could further strengthen the visibility of BiF_3_@PDA@HA nanoprobes in the fibrosis‐afflicted areas (**Figure** **5**a), resulting from the energy‐dependent attenuation characteristic of Bi. When the monochromatic energy decreased from 70 to 40 keV, the liver CT value of the targeted group increased up and peaked at 40 keV (109.67 ± 26.24 HU, maximum = 139.00 HU) (Figure 5b). Yet there was no significant variance in the control group over the monochromatic energy changing. Meanwhile, the targeted group's spectral HU curve also illustrated that the CT value increased with the decrease of monochromatic energy (Figure 5c, yellow line), showing a similar trend with the BiF_3_@PDA@HA phantom in vitro (Figure 2c). Nevertheless, due to the absence of contrast nanoagents, the adjacent muscle tissue did not have notable variance in CT value over monochromatic energy changing (Figure 5c, red line). Taken together, the fibrotic liver tissue accumulated with BiF_3_@PDA@HA nanoprobes possessed different X‐ray attenuation characteristics compared with other healthy soft tissue. These results may provide an excellent practical example for other imaging techniques using energy‐dependent differences, such as differential phase‐contrast X‐ray imaging^[^ ^31^ ^]^ and Raman microspectroscopy.^[^ ^32^ ^]^

#### 2.4.2 Targeting Performance of BiF_3_@PDA@HA in Liver‐Fibrosis Model

The phenomenal difference in CT values between fibrotic and healthy liver tissue was due to different enrichment of Bi, so the targeting ability of BiF_3_@PDA@HA nanoprobes toward liver fibrosis was next evaluated. Mice with liver fibrosis applied with BiF_3_@PDA@PEG nanoparticles were used as the non‐targeted group, in comparison to the targeted group described above. After the injection of BiF_3_@PDA@PEG, there was no obvious enhancement in hepatic parenchyma of the non‐targeted group (Figures S9 and S10, Supporting Information). In addition, compared to the targeted group, liver CT and ΔCT value of the non‐targeted group only increased slightly for 10–20 min post‐injection and then declined to baseline level (Figure S11, Supporting Information). Furthermore, CT value of the non‐targeted group was statistically significantly lower than that of the targeted group at each time point after injection (Table 1). These results clearly indicated that the favorable targeting performance of BiF_3_@PDA@HA nanoprobes was beneficial for the diagnosis of liver fibrosis via spectral CT.

### 2.5 Biocompatibility Assessment

All healthy mice injected with BiF_3_@PDA@HA well tolerated the nanoprobes without any short‐term (3 days) or long‐term (60 days) adverse effects as per hematological indices as well as histological observation of heart, liver, spleen, lung, and kidney (Figure S12, Supporting Information). Moreover, there was no evident weight difference between the BiF_3_@PDA@HA‐treated and phosphate‐buffered saline (PBS)‐treated mice over 60 days (Figure S13, Supporting Information). These results suggested the outstanding biocompatibility of BiF_3_@PDA@HA nanoprobes.

## 3 Conclusion

In conclusion, an energy‐dependent attenuation strategy was adopted to diagnose liver fibrosis accurately. BiF_3_@PDA@HA nanoprobes attenuated specifically depending on the level of X‐ray energy and showed characteristic performance correspondingly in monoenergetic imaging via spectral CT. As BiF_3_@PDA@HA nanoprobes could accumulate in fibrosis‐afflicted regions in liver and remarkably produced enhancement on 70 keV monoenergetic images, the fibrotic livers could be successfully differentiated from healthy ones, further achieving accurate diagnosis of liver fibrosis. Furthermore, due to the energy‐dependent attenuation of Bi, the diseased areas accumulated with BiF_3_@PDA@HA nanoprobes could be more conspicuous by selecting more suitable monochromatic energy via spectral CT. Especially, CT value of the fibrotic‐afflicted areas reached a maximum on 40 keV monoenergetic images (109.67 ± 26.24 HU) and was approximately 30 HU higher than the CT value at 70 keV (79.67 ± 11.57 HU). This energy‐dependent attenuation strategy was not only highly inspiring for other imaging techniques, but also ignited a novel approach to exploiting interdisciplinary efforts for accurate diagnosis of diseases.

## 4 Experimental Section

##### Materials

Bismuth nitrate (Bi[NO_3_]_3_·5H2O) and ethylene glycol (EG) were purchased from Adamas‐Beta (Shanghai, China), NH2‐(PEG) n‐SH (MW 2000 Da) from To Yong Bio (Shanghai, China), and Fluorescein isothiocyanate (FITC) isomer I from Sigma‐Aldrich (Shanghai, China). All other solvents and reagents were purchased from Aladdin Co. Ltd. (Shanghai, China). Deionized water was used for all experiments.

##### Characterizations

SEM (Hitachi S‐48000, Japan) and TEM (JEOL JEM‐2100 at 200 kV, JEOL Ltd., Japan) were used for morphological characterization. Element mapping was performed on a field emission Magellan 400 microscope. Powder XRD patterns were obtained using a Japanese Rigaku D/MAX‐2250V diffractometer with graphite‐monochromatized Cu K*α* radiation. FT‐IR spectroscopy was performed on a TENSOR II FT‐IR spectrophotometer (Bruker Corporation) by KBr. Dynamic light scattering tests were conducted on a Nicomp380 Z3000 SOP analyzer (Particle Sizing Systems). BiF_3_@PDA@HA nanoprobes were prepared using different concentrations (10 000, 5000, 2500, 1250, and 625 *μ*g mL^−1^) of Bi in the test for their CT imaging ability. The CT measurements were conducted on a clinical CT instrument (GE discovery CT750 HD).

##### Synthesis of Targeted Nanoprobes and Non‐targeted Nanoparticles

1. BiF_3_@PDA@HA nanoprobes: First, Bi(NO_3_)_3_·5H2O (1 mm) was dissolved in 10 mL of EG, followed by the addition of 25 mL of EG containing NH_4_F (24 mm). The solution was incubated for 30 min at 303 K with periodic stirring. After centrifugation, the products were washed twice with deionized water. The resulting BiF_3_ nanomaterials were then dispersed in 200 mL of deionized water. To convert BiF_3_ into hydrophilic BiF_3_@PDA nanoparticles, 400 mg of dopamine was added to the above solution and stirred vigorously for 30 min. Next, 500 µL of ammonium hydroxide was added into the system dropwise and stirred for 12 h. Then, the ensuing products BiF_3_@PDA nanoparticles were dispersed into 20 mL of deionized water. To conjugate HA onto the nanoparticles, 160 mg of sodium hyaluronate was subsequently added. After stirring for 30 min, 40 µL of ammonium hydroxide was added to the system. After vigorous stirring for 24 h, resulting products were centrifuged and washed with deionized water, and then dispersed in 20 mL of deionized water. Finally, liver‐fibrosis‐targeting BiF_3_@PDA@HA nanoprobes were successfully synthesized.
2. BiF_3_@PDA@HA nanoparticles: To acquire BiF_3_@PDA@PEG nanoparticles, all the steps, except the conjugation with PEG (160 mg), were the same as described above.

##### Cell Experiments

1. Cellular uptake experiment: Briefly, to label FITC onto nanoparticles, 0.1 mg of FITC were dissolved into 1 mL deionized water with 1 mg BiF_3_@PDA@HA and BiF_3_@PDA@PEG, respectively, in complete darkness. Then the solution was magnetically stirred vigorously at room temperature for 24 h. Hepatic stellate cells (HSC‐T6) were cultured with a specialized culture medium (both from Guangzhou Jennio Biotech Co., Ltd., China) in the CLSM‐special cell culture dish at 37 °C under 5% CO_2_. The solution containing labeled nanoparticles was centrifuged, washed thrice with phosphate‐buffered saline (PBS) (HyClone, USA), resuspended in the specialized culture medium for HSCs (Bi at 100 µg mL^−1^), and incubated with HSCs for 2 and 4 h. After being washed by PBS thrice to remove the free nanoparticles, the cells were stained with Hoechst 33342 (Beyotime, China) and observed under a confocal microscope in the DAPI (blue) and FITC (green) channels via CLSM (Nikon A1 + R‐980).
2. CD44 receptor blockade examination (competitive test): After applying an excess amount of HA (5 mg mL^−1^),^[^ ^33^ ^]^ the HSCs were treated with FITC‐labeled BiF_3_@PDA@HA nanoprobes for 2 and 4 h.
3. Cell toxicity assessment: Mouse liver parenchymal cell line (AML12) was obtained from the Shanghai Cell Bank, Chinese Academy of Science. The AML12 were cultured in mouse hepatocyte‐specialized medium (Shanghai Yaji Biotechnology Co., Ltd., Shanghai, China) at 37 °C under 5% CO_2_. To determine the cytotoxicity of BiF_3_@PDA@HA and BiF_3_@PDA@PEG, the cells were incubated with the respective nanoparticles for 24 h, and their viability was evaluated by the CCK8 assay (Dojindo Laboratories, Japan) according to the manufacturer's instructions. The cell viability was evaluated by the CCK8 assay (Dojindo Laboratories, Japan) according to the manufacturer's instructions. Absorbance at absorption wavelength of 450 nm was measured by the microplate reader (Bio‐TekELx800, USA).

##### Animal Experiments

All animal experiments were performed following the guidelines and protocols approved by the Institutional Animal Care and Use Committee of Fudan University (accreditation number: 202006011Z).

1. CT imaging experiment: C57BL/6 mice (Charles River, China) were randomly divided into the following groups: a) control group: healthy mice injected with BiF_3_@PDA@HA, b) targeted group: liver fibrosis model mice injected with BiF_3_@PDA@HA, and c) non‐targeted group: liver fibrosis model mice injected with BiF_3_@PDA@PEG. Liver fibrosis model mice were induced by feeding the mice with methionine/choline‐deficient (MCD) diet^[^ ^34^ ^]^ (SYSE Bio‐tech Co., Ltd., Jiangsu, China) for 12 weeks. The healthy controls were fed by regular diet. The animals were injected with BiF_3_@PDA@HA nanoprobes or BiF_3_@PDA@PEG nanoparticles via the intravenous route. After imaging, all the mice sacrificed by euthanasia and their liver were dissected and fixed in 4% paraformaldehyde for 24 h. After embedding in paraffin, the tissues were cut into 5–7 µm thick sections and stained with Sirius red, hematoxylin–eosin (H&E) and Masson's trichrome using standard protocols.
2. In vivo toxicity evaluation: The BiF_3_@PDA@HA (40 mg Bi per kg body weight) or PBS was injected intravenously into the normal ICR mice (Charles River, Beijing, China). After 3 and 60 days of injection, the mice were sacrificed. Moreover, the main organs, including heart, liver, spleen, lung, and kidney, were harvested for H&E staining to assess toxicity. Routine blood tests were conducted at Huashan Hospital, Fudan University. In addition, body weight of each animal was recorded for 60 days.

##### Computed Tomography Scanning

Conventional CT and spectral CT (Take gemstone spectral CT as an example) scans were performed on anesthetized mice placed in a supine position using a 64‐detector CT scanner (GE Discovery CT750 HD, GE Medical Healthcare, Wisconsin, USA). The polychromatic images were acquired from conventional CT parameters: tube voltage = 120 kVp; slice thickness = 5 mm. Monochromatic images and spectral HU curves were obtained on spectral CT scanning with following parameters: tube voltage = fast switching between 80 and 140 kVp; slice thickness = 0.625 mm; energy range = 40–140 keV. The scanning parameters of conventional CT and spectral CT were similar in the following aspects: field of view = 50 cm, window width = 350, and window level = 25. The images were processed using GSI Viewer (ADW4.7; GE Healthcare, Wisconsin, USA) in the pseudo‐color mode Sokoloff.

##### Statistics Analysis

1. Preprocessing and data analysis: The spectral HU curve was plotted after normalization (CT_water_ = 0). CT values of the liver parenchyma were measured by an experienced radiologist, who was blinded to the study. Regions‐of‐interest were manually drawn in circular or ellipse shape to encompass as much of the hyper‐enhanced portion as possible (mean pixel number 3, range 2–10) from three contiguous slices. Areas of focal changes of the parenchymal density, as well as large vessels and prominent artifacts, if any, were carefully avoided.
2. Data presentation, sample size, and statistical methods: Data were reported as mean ± standard error, and compared by two‐way ANOVA followed by Turkey post hoc test. *p* < 0.05 was considered the statistically significant level. Sample size (*n*) was three for each statistical analysis.
3. Software used for statistical analysis: GraphPad Prism 6.0 (GraphPad Software, La Jolla, CA) was used for all statistical analyses. **Table** **1**.

## Conflict of Interest

The authors declare no conflict of interest.

## Supporting information

Supporting Information

## Data Availability

Research data are not shared.

## References

[1] S. K. Asrani , H. Devarbhavi , J. Eaton , P. S. Kamath , J. Hepatol. 2019, 70, 151. 30266282 10.1016/j.jhep.2018.09.014

[2] M. Ge , H. Liu , Y. Zhang , N. Li , S. Zhao , W. Zhao , Y. Zhen , J. Yu , H. He , R. G. Shao , Br. J. Pharmacol. 2017, 174, 1147. 28257144 10.1111/bph.13766 PMC5406384

[3] Z. C. Jian , J. F. Long , Y. J. Liu , X. D. Hu , J. B. Liu , X. Q. Shi , W. S. Li , L. X. Qian , World J. Clin. Cases 2019, 7, 1122. 31183343 10.12998/wjcc.v7.i10.1122 PMC6547320

[4] H. Hagström , P. Nasr , M. Ekstedt , U. Hammar , P. Stål , R. Hultcrantz , S. Kechagias , J. Hepatol. 2017, 67, 1265. 28803953 10.1016/j.jhep.2017.07.027

[5] K. Zhang , Y. Han , Z. Hu , Z. Zhang , S. Shao , Q. Yao , L. Zheng , J. Wang , X. Han , Y. Zhang , T. Chen , Z. Yao , T. Han , W. Hong , Theranostics 2019, 9, 3622. 31281502 10.7150/thno.32935 PMC6587170

[6] Y. Lurie , M. Webb , R. Cytter‐Kuint , S. Shteingart , G. Z. Lederkremer , World J. Gastroenterol. 2015, 21, 11567. 26556987 10.3748/wjg.v21.i41.11567 PMC4631961

[7] a) X. Meng , H. Zhang , M. Zhang , B. Wang , Y. Liu , Y. Wang , X. Fang , J. Zhang , Z. Yao , W. Bu , Adv. Sci. 2019, 6, 1901214; 10.1002/advs.201901214 PMC6891899 31832312

[8] a) P. Y. Jung , E. J. Park , H. Shim , J. Y. Jang , K. S. Bae , S. Kim , Int. J. Surg. 2020, 77, 146;. 32198099 10.1016/j.ijsu.2020.03.022

[9] a) J. E. Burns , J. Yao , R. M. Summers , Radiology 2017, 284, 788;. 28301777 10.1148/radiol.2017162100 PMC5584647

[10] R. N. K. Bismark , R. Frysch , S. Abdurahman , O. Beuing , M. Blessing , G. Rose , Z. Med. Phys. 2020, 30, 40. 31831207 10.1016/j.zemedi.2019.10.002

[11] E. Pessis , R. Campagna , J. M. Sverzut , F. Bach , M. Rodallec , H. Guerini , A. Feydy , J. L. Drapé , Radiographics 2013, 33, 573. 23479714 10.1148/rg.332125124

[12] C. H. McCollough , S. Leng , L. Yu , J. G. Fletcher , Radiology 2015, 276, 637. 26302388 10.1148/radiol.2015142631 PMC4557396

[13] a) H. Ding , B. Zhao , P. Baturin , F. Behroozi , S. Molloi , Med. Phys. 2014, 41, 101901; 25281953 10.1118/1.4894724 PMC4281076

[14] H. W. Goo , J. M. Goo , Korean J. Radiol. 2017, 18, 555. 28670151 10.3348/kjr.2017.18.4.555 PMC5447632

[15] a) X. Lu , Z. Lu , J. Yin , Y. Gao , X. Chen , Q. Guo , Quant. Imaging Med. Surg. 2019, 9, 188;. 30976543 10.21037/qims.2018.11.12 PMC6414759

[16] D. P. Cormode , P. C. Naha , Z. A. Fayad , Contrast Media Mol. Imaging 2014, 9, 37. 24470293 10.1002/cmmi.1551 PMC3905628

[17] J. Hainfeld , S. Ridwan , F. Stanishevskiy , H. Smilowitz , Sci. Rep. 2020, 10, 15627. 32973267 10.1038/s41598-020-72268-0 PMC7515899

[18] Y. Dong , M. Hajfathalian , P. Maidment , J. Hsu , P. Naha , S. Si‐Mohamed , M. Breuilly , J. Kim , P. Chhour , P. Douek , H. Litt , D. Cormode , Sci. Rep. 2019, 9, 14912. 31624285 10.1038/s41598-019-50332-8 PMC6797746

[19] M. A. Shahbazi , L. Faghfouri , M. P. A. Ferreira , P. Figueiredo , H. Maleki , F. Sefat , J. Hirvonen , H. A. Santos , Chem. Soc. Rev. 2020, 49, 1253. 31998912 10.1039/c9cs00283a

[20] Y. Cheng , H. Zhang , Chem. ‐ Eur. J. 2018, 24, 17405. 29876975 10.1002/chem.201801588

[21] a) W. Liao , P. Lei , J. Pan , C. Zhang , X. Sun , X. Zhang , C. Yu , S.‐K. Sun , Biomaterials 2019, 203, 1;. 30844678 10.1016/j.biomaterials.2019.03.001

[22] P. F. Fitzgerald , R. E. Colborn , P. M. Edic , J. W. Lambert , A. S. Torres , P. J. Bonitatibus , B. M. Yeh , Radiology 2016, 278, 723. 26356064 10.1148/radiol.2015150577 PMC4770942

[23] K. Takahashi , S. Murata , K. Fukunaga , N. Ohkohchi , World J. Gastroenterol. 2013, 19, 5250. 23983427 10.3748/wjg.v19.i32.5250 PMC3752558

[24] Z. Hu , F. Qin , S. Gao , Y. Zhen , D. Huang , L. Dong , Am. J. Transl. Res. 2018, 10, 1012. 29636890 PMC5883141

[25] a) K. S. Kim , W. Hur , S. J. Park , S. W. Hong , J. E. Choi , E. J. Goh , S. K. Yoon , S. K. Hahn , ACS Nano 2010, 4, 3005;. 20518553 10.1021/nn100589y

[26] P. Lei , R. An , S. Yao , Q. Wang , L. Dong , X. Xu , K. Du , J. Feng , H. Zhang , Adv. Mater. 2017, 29, 1700505. 10.1002/adma.201700505 28370594

[27] Y. Jin , D. Ni , L. Gao , X. Meng , Y. Lv , F. Han , H. Zhang , Y. Liu , Z. Yao , X. Feng , W. Bu , J. Zhang , Adv. Funct. Mater. 2018, 28, 1802656.

[28] C. Zhang , X. Q. Liu , H. N. Sun , X. M. Meng , Y. W. Bao , H. P. Zhang , F. M. Pan , C. Zhang , Int. Immunopharmacol. 2018, 63, 183. 30098497 10.1016/j.intimp.2018.08.005

[29] a) K. Zhang , X. Han , Z. Zhang , L. Zheng , Z. Hu , Q. Yao , H. Cui , G. Shu , M. Si , C. Li , Z. Shi , T. Chen , Y. Han , Y. Chang , Z. Yao , T. Han , W. Hong , Nat. Commun. 2017, 8, 144; 28747678 10.1038/s41467-017-00204-4 PMC5529527

[30] Z. Sun , C. K. C. Ng , A. Squelch , Quant. Imaging Med. Surg. 2019, 9, 6. 30788242 10.21037/qims.2018.09.11 PMC6351807

[31] K. Mechlem , T. Sellerer , M. Viermetz , J. Herzen , F. Pfeiffer , Phys. Med. Biol. 2020, 65, 065010. 31995518 10.1088/1361-6560/ab7106

[32] R. J. Swain , S. J. Kemp , P. Goldstraw , T. D. Tetley , M. M. Stevens , Biophys. J. 2008, 95, 5978. 18820234 10.1529/biophysj.108.136168 PMC2599825

[33] Z. Wang , S. Sau , H. O. Alsaab , A. K. Iyer , Nanomedicine 2018, 14, 1441. 29678787 10.1016/j.nano.2018.04.004 PMC6192858

[34] K. Tomita , T. Teratani , T. Suzuki , M. Shimizu , H. Sato , K. Narimatsu , Y. Okada , C. Kurihara , R. Irie , H. Yokoyama , K. Shimamura , S. Usui , H. Ebinuma , H. Saito , C. Watanabe , S. Komoto , A. Kawaguchi , S. Nagao , K. Sugiyama , R. Hokari , T. Kanai , S. Miura , T. Hibi , Hepatology 2014, 59, 154. 23832448 10.1002/hep.26604

