# Supplementary material for: Harnessing X‐Ray Energy‐Dependent Attenuation of Bismuth‐Based Nanoprobes for Accurate Diagnosis of Liver Fibrosis
Source: Adv Sci (Weinh). 2021 Mar 16;8(11):2002548. doi: 10.1002/advs.202002548 (PMC8188217; doi:10.1002/advs.202002548)
Supplement: Supplementary file 1 — Supporting Information [file ADVS-8-2002548-s001.pdf]

© 2021 Wiley-VCH GmbH

ADVANCED SCIENCE
Open Access

Supporting Information

for *Adv. Sci.,* DOI: 10.1002/advs.202002548

Harnessing X-ray Energy Dependent Attenuation of Bismuth-Based Nanoprobes for Accurate Diagnosis of Liver Fibrosis

Shiman Wu[1], Xianfu Meng[2,3], Xingwu Jiang[2], Yelin Wu[3], Shaojie Zhai[4], Xiaoshuang Wang[1], Yanyan Liu[2], Jiawen Zhang[1], Xinxin Zhao[5], Yan Zhou[5], Wenbo Bu[2,4]*, Zhenwei Yao[1]**

© 2021 Wiley-VCH GmbH

Supporting Information

Harnessing X-ray Energy Dependent Attenuation of Bismuth-Based Nanoprobes for Accurate Diagnosis of Liver Fibrosis

Shiman Wu[1], *Xianfu Meng*[2,3], *Xingwu Jiang*[2], *Yelin Wu*[3], *Shaojie Zhai*[4], *Xiaoshuang Wang*[1], *Yanyan Liu*[2], *Jiawen Zhang*[1], *Xinxin Zhao*[5], *Yan Zhou*[5*], *Wenbo Bu*[2,4]*, *Zhenwei Yao*[1]*

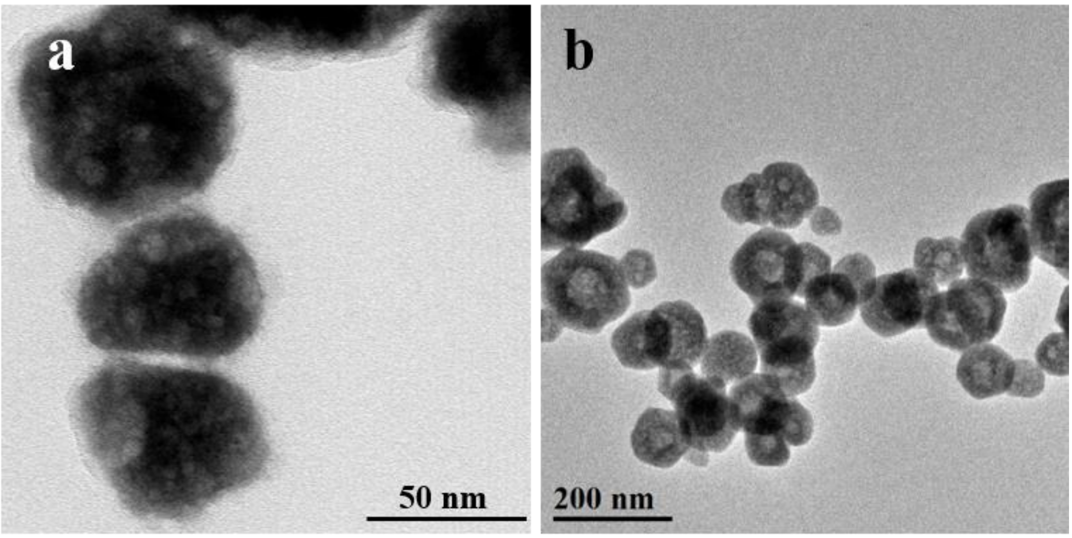

Figure S1. Transmission electron microscopy (TEM) images of a) BiF_3 and b) BiF_3@PDA@HA .

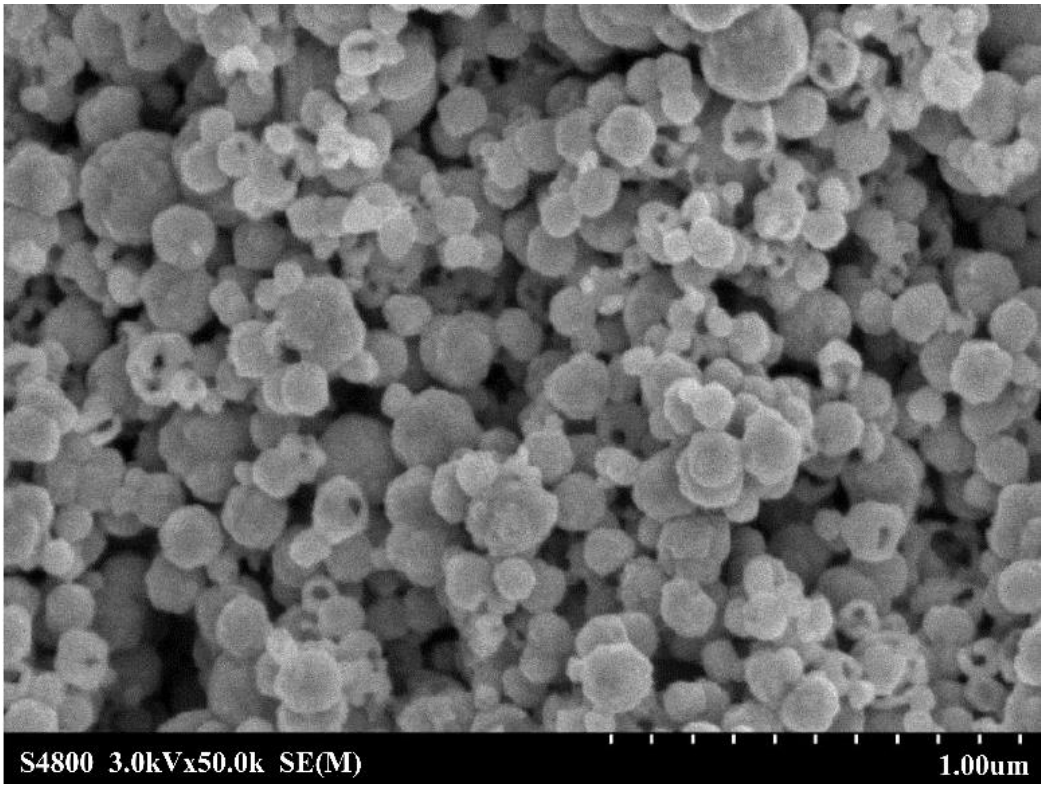

Figure S2. Scanning electron microscopy (SEM) images of BiF_3.

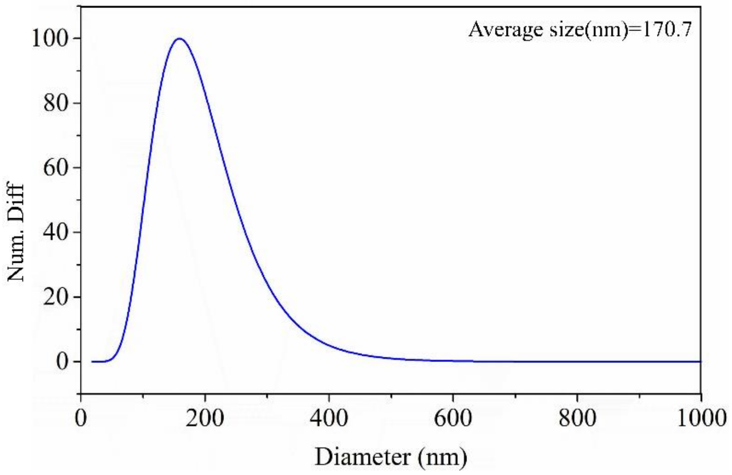

Figure S3. Dynamic light scattering(DLS) distribution curve of BiF_3@PDA@HA.

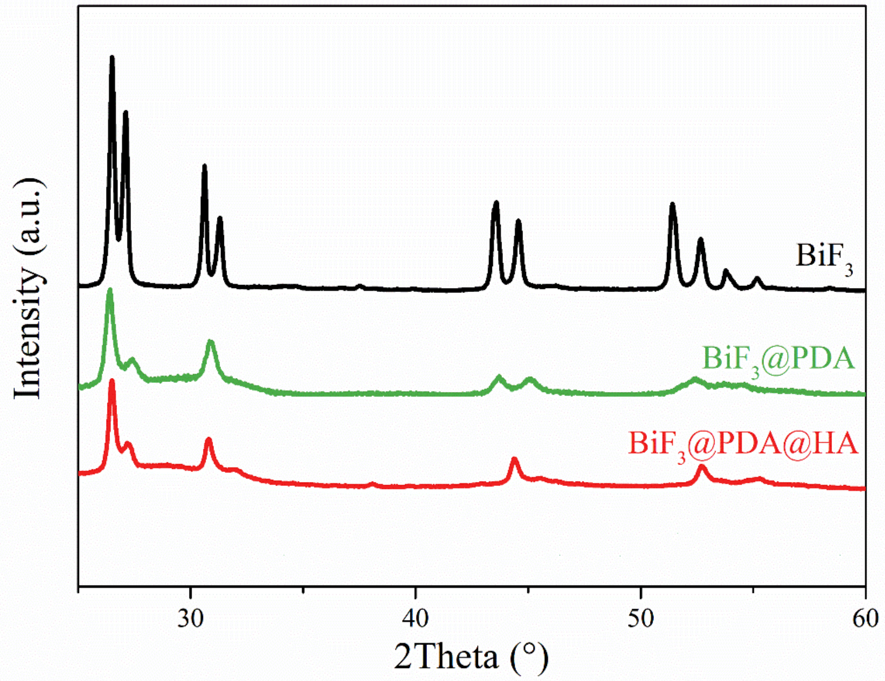

Figure S4. X-ray diffraction (XRD) patterns of BiF_3 (JCPDS No. 70-2407), BiF_3@PDA, and BiF_3@PDA@HA.

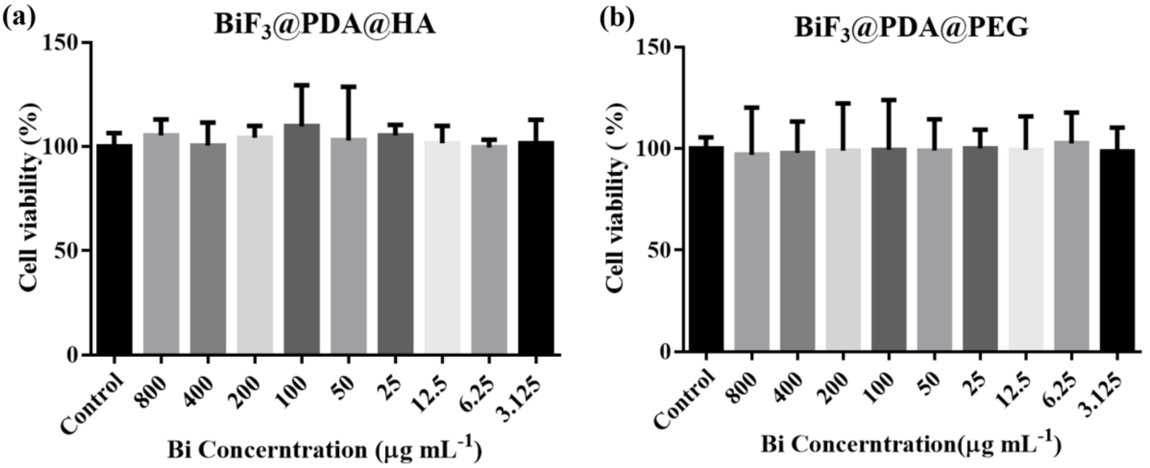

Figure S5. CCK8 assay of BiF_3@PDA@HA (a), and BiF_3@PDA@PEG (b) in AML cells for 24-hour co-incubation(n=6). Data was illustrated as mean± standard deviation.

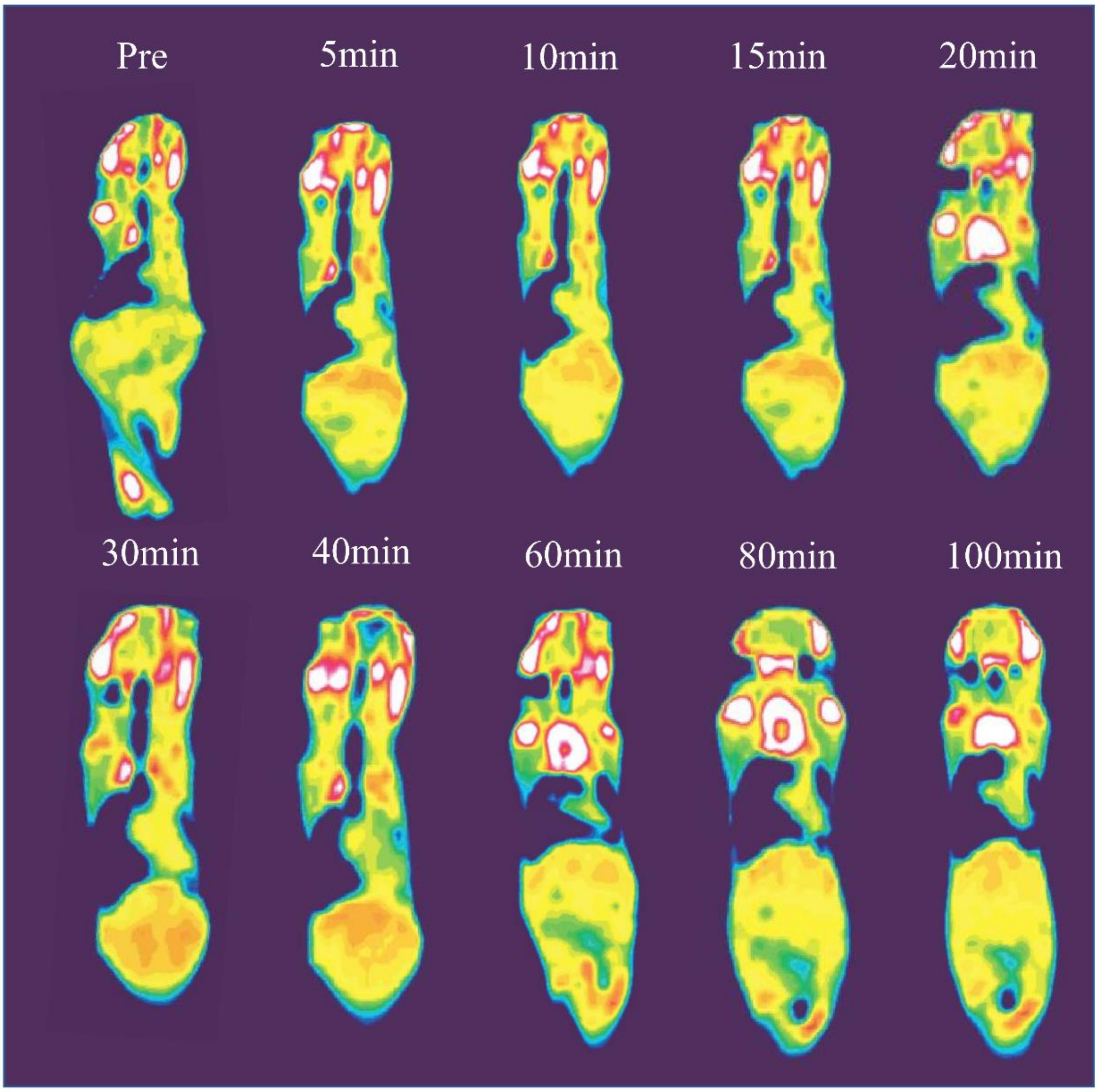

Figure S6. Representative computed tomography (CT) images of mice in the targeted group (liver fibrosis model mice injected with BiF_3@PDA@HA, n=3) before and after injection of BiF_3@PDA@HA in the coronal projection.

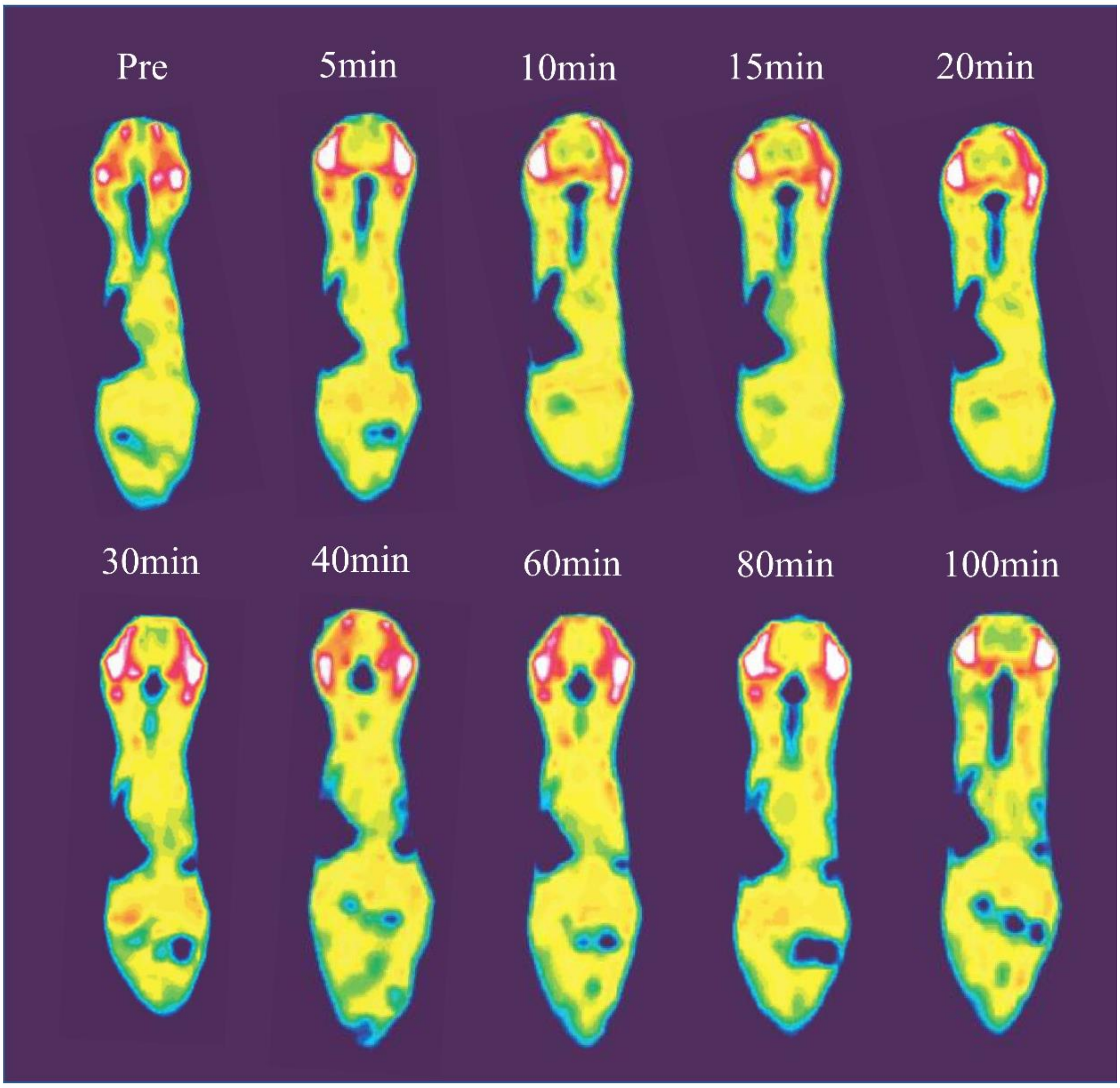

Figure S7. Representative spectral computed tomography (CT) images of mice in the control group(healthy mice injected with BiF_3@PDA@HA, n=3) before and after injection of BiF_3@PDA@PEG in the coronal projection.

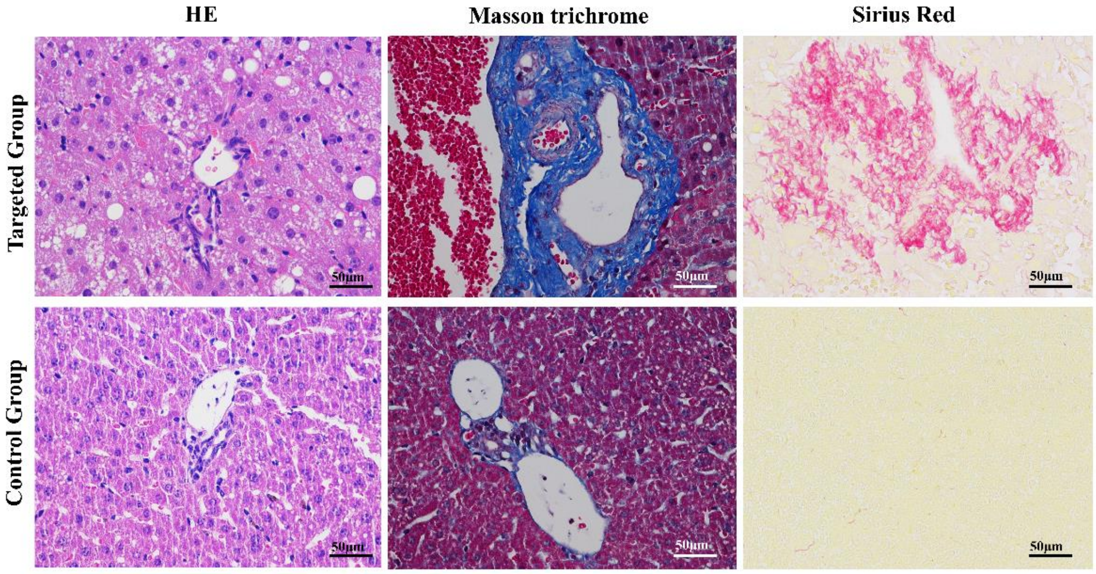

Figure S8. Representative histologic images (400 x) from mice models with liver fibrosis in the targeted group, as well as healthy mice in the control group.

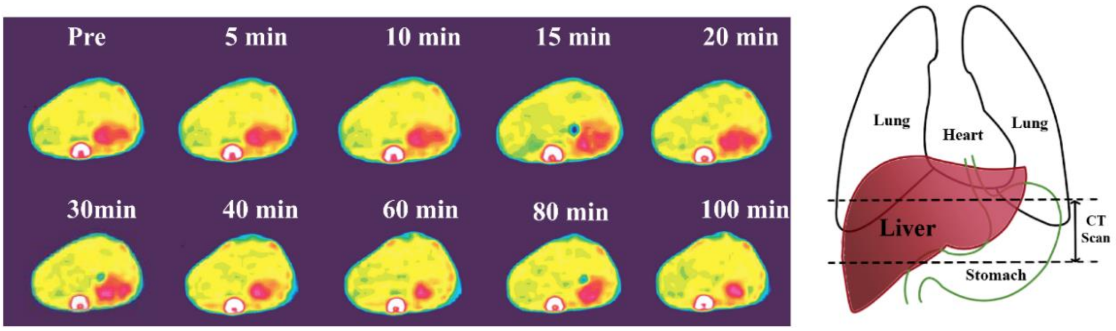

Figure S9. Representative spectral computed tomography(CT) images of the non-targeted group (liver fibrosis mice injected with BiF_3@PDA@PEG, n=3) pre- and post- injection of BiF_3@PDA@PEG. The schematic drawings illustrate the taken CT sections.

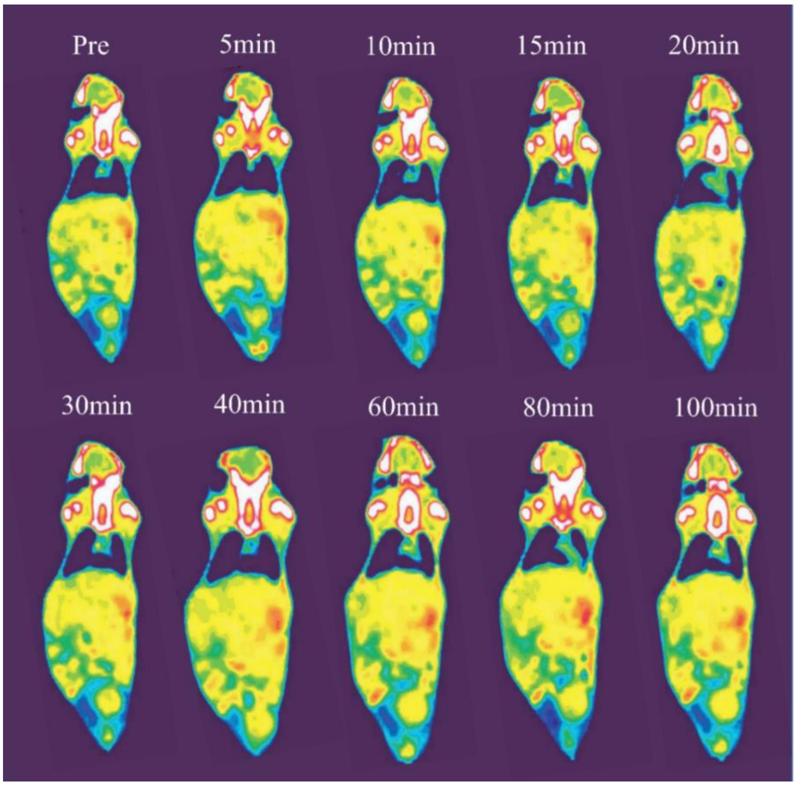

Figure S10. Representative spectral computed tomography(CT) images of mice in the non-targeted group (liver fibrosis mice injected with BiF_3@PDA@PEG, n=3) before and after injection of BiF_3@PDA@PEG in the coronal projection**.**

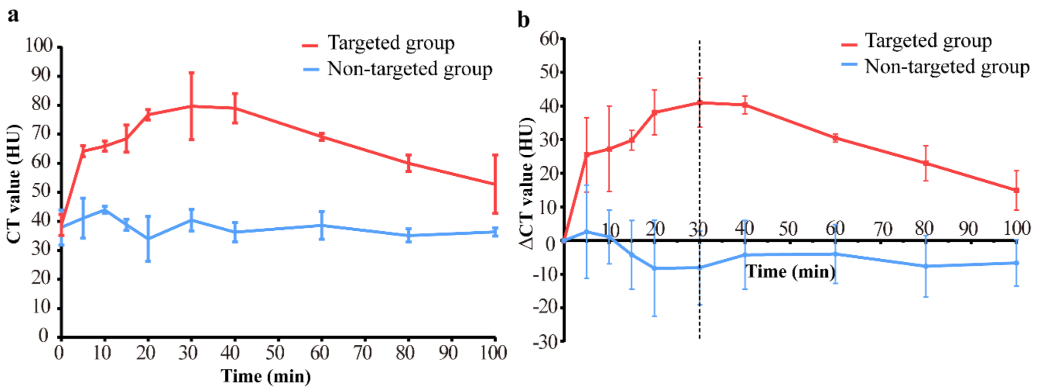

Figure S11. Dynamic time courses of the CT value (a) and CT value change (ΔCT value) (b) of hepatic parenchyma in targeted group (liver fibrosis mice with BiF_3@PDA@HA, n=3, red line) and non-targeted group (liver fibrosis model mice with BiF_3@PDA@PEG, n=3, blue line). Data was illustrated as mean± standard deviation. CT: Computed tomography; HU: Hounsfield unit.

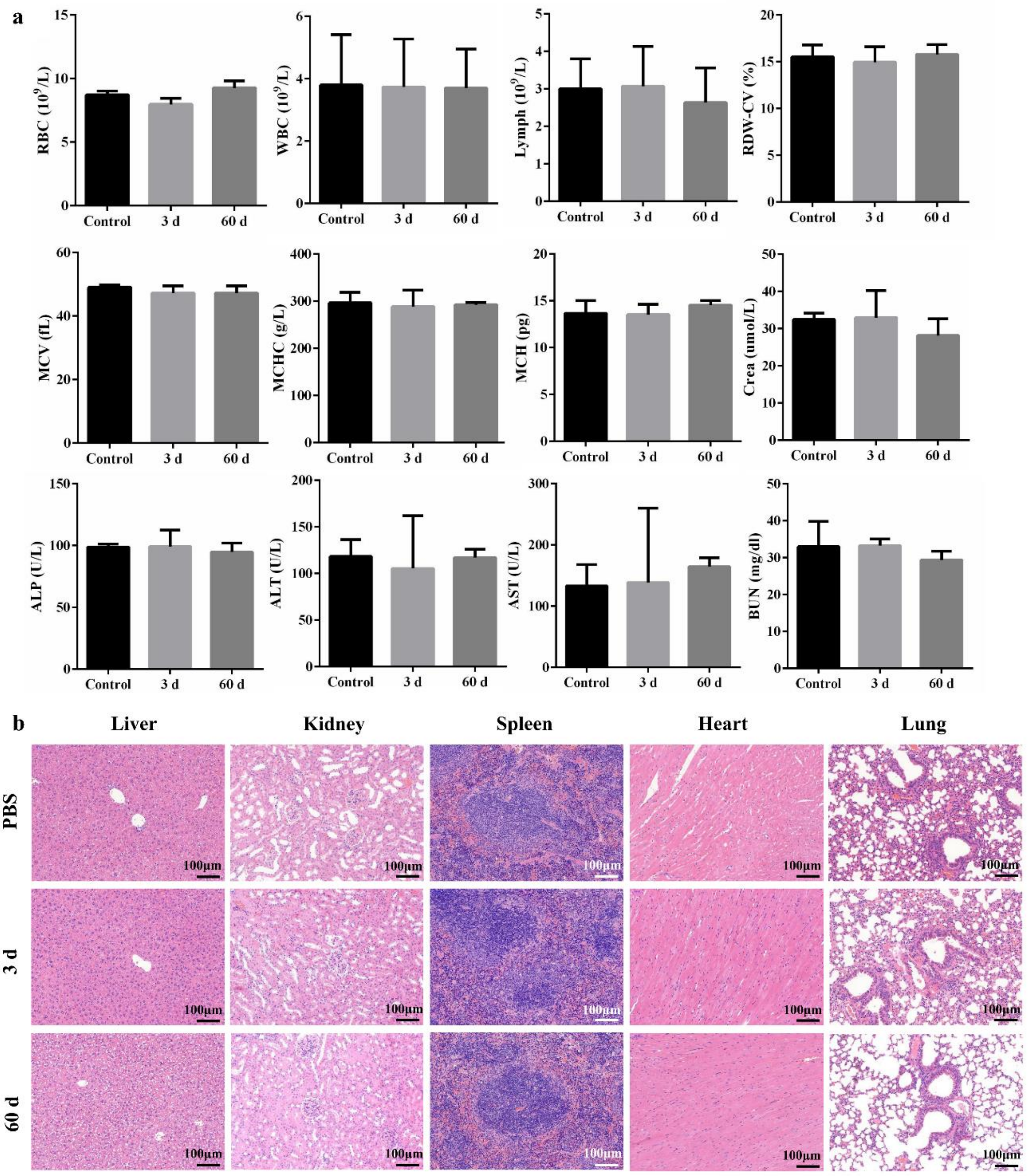

Figure S12. *In vivo* toxicity evaluation of BiF_3@PDA@HA. a) Hematological indices and b) hematoxylin and eosin (H&E) staining of major organs of ICR mice at 3 days and 60 days post-injection of BiF_3@PDA@HA(n=3). Data was illustrated as mean± standard deviation. Data was illustrated as mean± standard deviation.

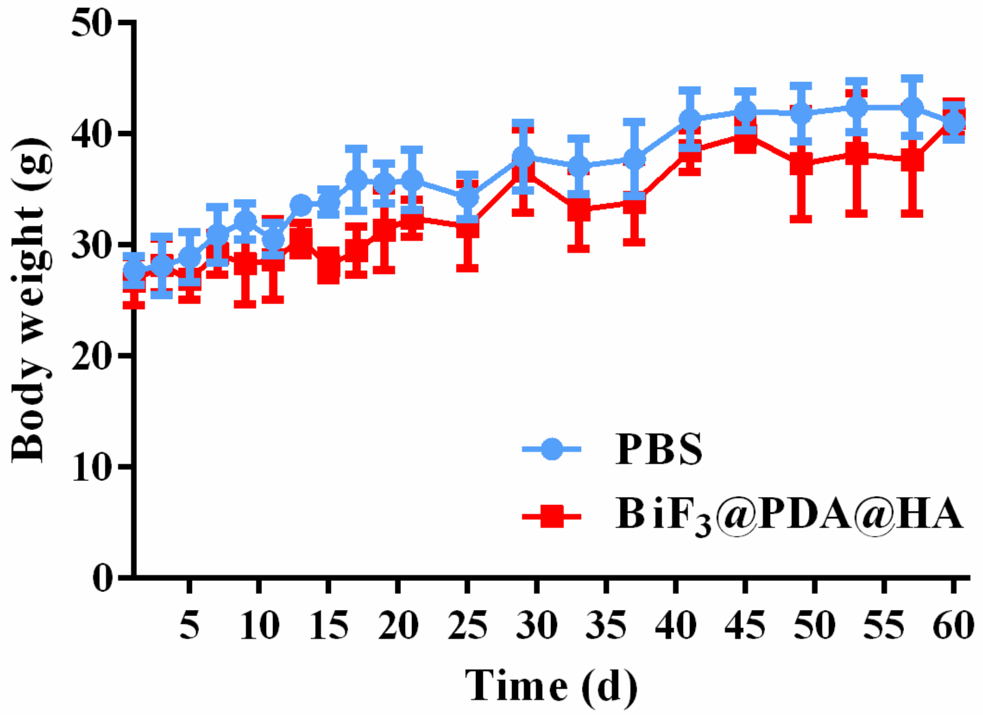

Figure S13. Body weight fluctuations in mice injected with BiF_3@PDA@HA nanoprobes (n=3) or treated with PBS (n=3) for 60 days. Data was expressed as mean± standard deviation.
